# Significance of Cardiac Troponins as an Identification Tool in COVID-19 Patients Using Biosensors: An Update

**DOI:** 10.3389/fmolb.2022.821155

**Published:** 2022-02-24

**Authors:** Yousef Rasmi, Osama F. Mosa, Shahriar Alipour, Nadia Heidari, Farzaneh Javanmard, Ali Golchin, Shiva Gholizadeh-Ghaleh Aziz

**Affiliations:** ^1^ Cellular and Molecular Research Center, Urmia University of Medical Sciences, Urmia, Iran; ^2^ Department of Biochemistry, School of Medicine, Urmia University of Medical Sciences, Urmia, Iran; ^3^ Public Health Department, Health Sciences College at Lieth, Umm Al Qura University, Mecca, Saudi Arabia; ^4^ Biochemistry Department, Bukhara State Medical Institute Named After Abu Ali ibn Sino, Bukhara, Uzbekistan; ^5^ Department of Clinical Biochemistry, Metabolic Disorders Research Center, Golestan University of Medical Sciences, Gorgan, Iran; ^6^ Department of Pathology, Urmia University of Medical Science, Urmia, Iran; ^7^ Nephrology and Kidney Transplant Research Center, Clinical Research Institute, Urmia University of Medical Sciences, Urmia, Iran

**Keywords:** cardiovascular disease, troponin, COVID-19, biosensor, SARS-CoV-2, diagnostic value

## Abstract

Coronavirus disease 2019 (COVID-19) has rapidly developed as a global health emergency. Respiratory diseases are significant causes of morbidity and mortality in these patients with a spectrum of different diseases, from asymptomatic subclinical infection to the progression of severe pneumonia and subsequent acute respiratory distress syndrome. Individuals with cardiovascular disease are more likely to become infected with SARS-CoV-2 and develop severe symptoms. Hence, patients with underlying cardiovascular disease mortality rate are over three times. Furthermore, note that patients with a history of cardiovascular disease are more likely to have higher cardiac biomarkers, especially cardiac troponins, than infected patients, especially those with severe disease, making these patients more susceptible to cardiac damage caused by SARS-2-CoV. Biomarkers are important in decision-making to facilitate the efficient allocation of resources. Viral replication in the heart muscle can lead to a cascade of inflammatory processes that lead to fibrosis and, ultimately, cardiac necrosis. Elevated troponin may indicate damage to the heart muscle and may predict death. After the first Chinese analysis, increased cardiac troponin value was observed in a significant proportion of patients, suggesting that myocardial damage is a possible pathogenic mechanism leading to severe disease and death. However, the prognostic performance of troponin and whether its value is affected by different comorbidities present in COVID-19 patients are not known. This review aimed to assess the diagnostic value of troponin to offer insight into pathophysiological mechanisms and reported new assessment methods, including new biosensors for troponin in patients with COVID-19.

## 1 Introduction

Coronavirus disease 2019 (COVID-19) is an emerging outbreak from Wuhan City, China, caused by the severe acute respiratory syndrome coronavirus 2 (SARS-CoV-2) (Malik et al., 2020). In 15% of infected patients, the clinical course of this pathology is complicated by the development of severe forms of interstitial pneumonia, which may lead to acute respiratory distress syndrome (ARDS), multi-organ failure (MOF), and death (Mattiuzzi and Lippi, 2020). Although the cardiovascular system did not appear to be particularly affected by the virus at the start of the pandemic, other studies found that patients with a history of cardiovascular disease or cardiovascular risk factors had higher mortality rates than those without a history of cardiovascular disease (Akhmerov and Marbán, 2020; Bansal, 2020).

Emerging literature has reported that 7%–28% of COVID-19 patients had developed an acute cardiac injury, eventually causing more complications and mortality (Wang et al., 2020a; Guo et al., 2020). In addition, several studies have shown that COVID-19 patients who did not have heart disease prior to infection may have heart problems (Kim et al., 2020; Zeng et al., 2020; Zhou, 2020). Early diagnosis of heart disease in these patients is possible by measuring cardiac troponin as the gold standard marker of myocardial damage (Park et al., 2017). The most recent international guidelines recommend cardiac Troponin I (hs-cTnI) and T (hs-cTnT) testing, which are particularly sensitive for diagnosis of myocardial injury and acute myocardial infarction (MI). Cardiac troponins have gradually gained greater clinical importance in the diagnosis, treatment, and prognosis of patients with cardiovascular disease (Park et al., 2017; Clerico et al., 2019). However, there are few studies that focused on the role and concentration of highly sensitive cardiac troponins in patients diagnosed with COVID-19.

This study aimed to evaluate the diagnostic value of troponin to offer insight into pathophysiological mechanisms and reported new assessment methods, including new biosensors for troponin in patients with COVID-19.

## 2 Overview of SARS COV-2

Over the past two decades, outbreaks of different viral diseases have occurred, including the human immunodeficiency virus (HIV/AIDS) from 1981 to present, severe acute respiratory syndrome coronavirus (SARS-CoV-1) during 2002–2004, influenza A virus subtype H1N1 (A/H1N1) during 2009–2010, the Middle East respiratory syndrome coronavirus (MERS-CoV) in 2012-present, Ebola virus disease (EVD) [which was known as Ebola Hemorrhagic Fever (EHF)] during 2013–2016, Zika virus (ZIKV) in 2015, and the most recent and important outbreak in 2020, which is called severe acute respiratory syndrome coronavirus 2 (SARS-CoV-2). Definitively, SARS-CoV-2 has an outstanding genetic symmetry (96.2%) with the bat coronavirus RaTG13, which was secured from bats in Yunnan in 2013, but there are some discrepancies in the origin of SARS-CoV-2, which needs to be further investigated (Paraskevis et al., 2020). Incipiently, SARS-CoV-2 prompted a series of acute abnormal respiratory diseases in Wuhan, Hubei Province, China, in December 2019. After a few months, the World Health Organization (WHO) termed the SARS-CoV-2 infection disease as coronavirus disease 2019 (COVID-19) and declared this ongoing outbreak on January 30, 2020 (Majumder and Minko, 2021). COVID-19, the cause of the current global pandemic, is an infectious disorder caused by a lately discovered RNA virus called SARS-CoV-2 (Basiri et al., 2021). The main concerns of COVID-19 are its rapid spread and high mortality rate (Basiri et al., 2021). Many countries have also been forced to adopt a virtual work environment and to impose repeated lockdowns. Based on the Worldometer site data, since August 9, 2021, more than 203,500,000 coronavirus cases worldwide have been reported (https://www.worldometers.info/).

After the COVID-19 outbreak, the race among different companies to promote effective vaccines and therapeutics to treat this mysterious disease, as well as diagnostic tests, has begun, with many trials underway (Basiri et al., 2021; Golchin, 2021). Reputably, fast and reliable detection of SARS-CoV-2 in potential patients is crucial to control this outbreak in hospitals and societies (To et al., 2020; Zhai et al., 2020). The current main screening tests for COVID-19 patients include real-time (r) reverse-transcription (RT) polymerase chain reaction (PCR) (rRT-PCR) and similar modified molecular tests to detect the presence of specific genetic material of the virus (To et al., 2020; Zhai et al., 2020). The positive rate of rRT-PCR for oropharyngeal swabs of COVID-19 patients has been reported to be 53.3%–71% in different studies (Fang et al., 2020; Zhang et al., 2020). However, the RT-PCR results ordinarily after 2–8 days become positive (Huang et al., 2020). However, several studies emphatically suggest utilizing viral Ig M and Ig G serological tests and computerized tomography (CT) scans of the chest to confirm PCR results (Fang et al., 2020; Zhai et al., 2020; Zhang et al., 2020). Different therapeutic approaches referring to scientific reports have been considered. To date, there are no prevailing data from randomized controlled clinical trials to support any particular anti-SARS-CoV-2 agents for COVID-19 patients; however, lopinavir (LPV), favipiravir, remdesivir, chloroquine, and hydroxychloroquine as antiviral agents, convalescent plasma and CR3022 as antibodies, and dexamethasone, prednisone, methylprednisolone, and hydrocortisone as corticosteroids have been considered in COVID-19 treatment guidelines (Zhai et al., 2020; Golchin, 2021). Moreover, several cell-based therapy products such as mesenchymal stem cells, natural killer cells, dendritic cells, and exosomes have been reported to have positive results for treating severe COVID-19 patients in some clinical trials (Basiri et al., 2021; Golchin, 2021). Furthermore, several vaccines have been developed based on conventional and innovative vaccine development models that demonstrate promising antibody responses to help prevent people from getting infected with SARS-CoV-2 (Soiza et al., 2021). Several vaccines have been approved for emergency use authorization, such as Pfizer-BioNTech, Moderna, Johnson and Johnson’s Janssen, Sinopharm, and AstraZeneca.

The COVID-19 outbreak is a potent warning of the current challenge of emerging and re-emerging infectious pathogens and the demand for regular surveillance, rapid diagnosis, and extensive investigation to explain the basic biology of viruses and the physiopathology of their causative diseases.

## 3 Biomarker Changes in COVID-19

Detection of biomarkers is important in classifying patients at risk of developing COVID-19, and their molecular classification is vital to improve treatment and diagnosis. In this part, an updated summary of routine biomarker levels in COVID-19-positive and -negative patients is provided with an in-depth look at the potential application of these biomarkers for diagnosis, prognosis, and treatment. Besides, a classification of different biomarkers based on the organs involved is shown in Table 1.

**TABLE 1:** The future Biomarkers and diagnostic utility against COVID-19.

Biomarkers	Organ/System involved	Type of biomarkers	Role/Effect	Step of disease (mild-severe-critical)	Ref
1-cytokines:(IL-6, IL-10, IL-1R, MCP-1, TNF-alpha)	Inflammation system (serum)	Immunological	Role in severity:(IL-6, IL-1R, TNF are increased)	Severe	Lippi and Plebani (2020a), Hu et al. (2020)
2-chemokines (CXCL8, CXCL9, CXCL10)			GFs were significantly higher in fatal than severe and/or mild but not correlated to disease severity	Fatal	Xu et al. (2020)
3-procalcitonin			Prognosis role: risk factor of in-hospital mortality	Severe	Lippi and Plebani (2020a), Hu et al. (2020)
4-neopterin			Prognostic role: higher in severe COVID-19 disease patients	Severe	Ozger et al. (2021)
1-lymphocyte counts (LYM)	Hematological (serum)	Immunological	Predictor of prognosis: LC decrease	Severe	Tan et al. (2020)
2-neutrophil counts (NØ)			Neutrophilia-induced lung injury in severe patients	Severe	Wang et al. (2020b)
3-neutrophil-to-lymphocyte ratio (NLR)			An independent risk factor of the in-hospital mortality, NLR increases	moderate-severe ARDS in severe COVID-19	Liu et al. (2020b), Ma et al. (2020)
4-neutrophil-to-CD8^+^ T cell ratio (N8R)			Powerful prognostic factors	Severe	Liu et al. (2020c)
5-eosinophil counts (EØ)			Was generally very low in the early stages of the disease in severe patient	Early stages/severe	Zheng et al. (2020)
6-platelet counts (PLT)			PLT decrease	Severe	Zheng et al. (2020)
7-platelet-to-lymphocyte ratio (PLR)			Had higher levels on admission	Severe	Simadibrata et al. (2020)
D-dimer levels	Coagulation (serum)	Biochemical	D-dimer increase (≥0.5 mg/L)		Garcia-Olivé et al. (2020)
Serum ferritin			Ferritin increase severity in hospitalized patients	Severe	Kappert et al. (2020)
Aspartate aminotransferase (AST)	Hepatic and metabolic	Biochemical	Severity and mortality diagnostic	Severe	Malik et al. (2021)
Alanine aminotransferase (ALT)			Elevated ALT (>40 IU/L)	Severe	Malik et al. (2021)
Lactate dehydrogenase (LDH)			LDH increase	Unclear	Yuan et al. (2020), Serrano-Lorenzo et al. (2021)
C-reactive protein			CRP increase	Severe	Kermali et al. (2020)
Cardiac troponin (cTn)	Cardiac Muscle	Biochemical	Severity and mortality increase		Henry et al. (2020b)
Creatinine proteinuria	Renal	Urine sample	Severity: Urea and creatinine increase	1. Severe	Cheng et al. (2020), Ouahmi et al. (2021)
				2. Moderate to severe	

Biomarkers are measurable parameters. For example, levels of gene expression and protein are evaluated as common or pathological diagnostic factors, whether available or mediated (Kraus, 2018; Caruso et al., 2021). Depending on the application, biomarkers can be classified as hazards that cause long-term sensitivity, pharmacodynamics/response, diagnosis, recurrence, analysis, monitoring, evaluation of the most effective biotherapeutic, predictive and safety markers. It is essential to differentiate between prognostic biomarkers that are valuable to recognize patients more likely to have a particular result individually from treatment and predictive biomarkers that contain a relationship of a treatment to control in patients with and without the biomarker.

Numerous prognostic biomarkers in COVID-19 that predict disease intensity have already been confirmed in clinical situations (Kermali et al., 2020; Caruso et al., 2021). A recent retrospective cohort study concluded that among biomarkers that discriminate between severe and non-severe patients are those related to changes in immune responses and its imbalance (Liu et al., 2020a; Qin et al., 2020a). Studies that review the role of cytokines in SARS and MERS have also specified an association between cytokine release syndrome (CRS) and their severity. Realizing their function in COVID-19 may support simplifying the plan of unique immunotherapies (Mahallawi et al., 2018).

Biomarkers associated with infectious diseases, such as inflammatory factors IL-2R, TNF*α*, and IL-6 and factors related to cell count, are evident in high proportions in more severe patients than milder ones (Qin et al., 2020b). In contrast, platelet count decreases in severe cases only (Lippi et al., 2020). The results of a meta-analysis reviewing six studies showed that interleukin-6 levels were significantly increased (2.9-fold) in severe COVID-19 cases compared to mild cases (Coomes and Haghbayan, 2020).

Factors related to inflammation as biomarkers may be beneficial in recognizing COVID-19 and distinguishing it from other viral pneumonia diseases. C-reactive protein (CRP) is an acute phase reactant protein that is increased in response to inflammation, simultaneously with a boost of other inflammatory cytokines associated with severity and mortality of COVID-19 patients (Ali, 2020). Another study conducted in Wuhan showed significantly higher CRP levels in severe COVID-19 patients than others (Qin et al., 2020c).

Procalcitonin, a factor formed by many kinds of cells in the body, is an extra inflammatory factor thought to be more particular for most bacterial infections. Some papers reported that levels of procalcitonin were related to the severity of COVID-19-positive individuals and can, as such, help to confirm COVID-19 in some cases (Hu et al., 2020). The results of a meta-analysis study showed that people with high procalcitonin had a five times higher risk of severe SARS-CoV-2 (Lippi and Plebani, 2020a).

Gene sequencing studies have shown that genetic changes related to chromosome 3 have been closely associated with the severity of infection to SARS-CoV-2 and hospitalization (Group, 2020; Initiative, 2020). Additional biomarkers associated with SARS-CoV-2 that are related to severity and mortality include cardiovascular biomarkers, of which cardiac troponin (cTn) is important (Zhou et al., 2020), or to the rate of chronic kidney diseases where a rise of creatinine amounts is detected in severe patients (Cheng et al., 2020). In addition to these medical biomarkers, there is now extensive literature on molecular factors that identifies the disease related to SARS-CoV-2 infection and that can be used to identify therapeutic targets.

Other studies have revealed considerably higher levels of kidney markers such as serum urea, creatinine, and markers of glomerular filtration rate in severe patients. In a study with a large sample (*n* = 701), it was found that raised serum creatinine levels on admission correlated with severity due to significant abnormalities in the coagulation pathway (Cheng et al., 2020).

Acute kidney damage (AKI), coronary artery and cerebrovascular disease, and lung tissue disorders such as COPD all occurred in severe COVID-19 cases and they were linked to leukocytosis, high creatinine kinase, and raised LDH and PT. Additionally, AKI is viewed as a critical sign of disease severity and is assessed using prognostic variables such as serum creatinine (sCr), urea, and cystatin C (Rasmi et al., 2021).

The results of this study and previous studies show that no particular biomarker will have the sensitivity and specificity to identify or reject COVID-19. There is a plan for sharing biomarkers and combining a merged reference standard for analyzing COVID-19 (Graziadio, 2020). This decision looks sensible when bringing up the biomarkers recognized by the current study, where low lymphocyte counts (LYM), eosinophil counts (EØ), neutrophil-to-lymphocyte 3-ratio (NLR), platelet counts, and platelet-to-lymphocyte ratio (PLR) are unlikely to segregate between respiratory infections and COVID-19. On the other hand, values of C-reactive protein, IL-6, and other biochemical factors such as lactate dehydrogenase, aspartate aminotransferase, and alanine aminotransferase exhibited high specificity against COVID-19 (Cheng et al., 2020; Kermali et al., 2020; Rasmi et al., 2021) (Table 1).

Angiotensin-converting enzyme 2 (ACE2) is a transmembrane receptor that converts angiotensin II to angiotensin (Wiese et al., 2021). It has been reported that ACE2 is expressed by different tissues, such as small intestine, colon, kidney, and heart. It is proved that ACE2 is expressed by cardiomyocytes, cardiac fibroblasts, and coronary artery endothelial cells (Herman-Edelstein et al., 2021). Notably, ACE2 is associated with developing hypertension and COVID-19 and has a harmful effect on renal tissues (Li et al., 2017; Bourgonje et al., 2020). Many studies have shown that SARS-CoV-2 can bind with ACE2 through the spike (S) protein and then enter host cells and subsequently infect cardiac cells, resulting in myocardial injury or death (Ni et al., 2020). However, acute renal damage and/or acute tubular necrosis may occur during SARS-CoV-2 infection, and based on the results mentioned in this section, it can be said that hypertension is the most common accompanying symptom observed in patients with COVID-19 (Rasmi et al., 2021). In this regard, another critical element is transmembrane serine protease 2 (TMPRSS2), which belongs to the serine protease family. TMPRSS2 is mainly expressed in the salivary gland, lung, thyroid, gastrointestinal tract, pancreas, kidney, and heart muscle (48). It seems that TMPRSS2 plays a vital role in coronavirus disease. Indeed, TMPRSS2 cleaves spike glycoprotein, facilitating viral entry (Peacock et al., 2021).

## 4 Troponin Structure and Function

Cardiac troponins have three subunits (Troponin C, Troponin T, and Troponin I). Troponin C, namely, TN-C or TnC, is categorized as a calcium-binding protein expressed from the TNNC1 gene in cardiac and skeletal muscle (Cheng and Regnier, 2016; Marston and Zamora, 2020a). Troponin I is known as the inhibitory subunit, which inhibits the interaction of myosin with actin. There are three different isoforms (the fast and slow skeletal isoforms and the cardiac-specific isoform) for Troponin I. Troponin T, the largest subunit including 288 amino acids (36 kDa), is responsible for cardiac contraction. Troponin T can be divided into different functional regions, such as the N-terminus, also known as the T1 region (interacts with tropomyosin), and the C-terminus, also known as the T2 region (Cheng and Regnier, 2016).

Cardiac Troponins I and T are considered biomarkers for myocardial injury expressed in cardiomyocytes. In physiological conditions, troponin is presented in very small to undetectable quantities in the blood; however, in pathological conditions such as myocardial injury, troponin is increased in the blood (Maynard et al., 2000; Silva et al., 2010). Based on findings, it seems that cardiac troponin is released into blood circulation during the first 24 h and mostly subside within 2 weeks (Katus et al., 1991). Several studies showed that cardiac troponins are the more sensitive and more specific marker for diagnosis of myocardial injury compared to other makers, such as CK-MB (Al-Hadi and Fox, 2009).

In humans, Troponin I is extracted in three isoforms: specific cardiac and fast and slow isoforms (Wade et al., 1990; Tiso et al., 1997). The cardiac isoform of TnI is stated absolutely in the heart (Bodor et al., 1995; Bhavsar et al., 2000; Dellow et al., 2001). The gene of cardiac TnI (TNNI3) is placed on the 19th chromosome (19q 13.4) and involves seven introns and eight exons (Bhavsar et al., 1996). The cTnI consists of five domains: C-terminal mobile domain, regulatory domain, inhibitory domain, IT-arm, and N terminal domain (Katrukha, 2013). At high levels of Ca^2+^, this domain interacts with a hydrophobic cleft on the surface of the TnC N-terminal domain, creating the third link region of hcTnI with TnC interaction of the regulatory domain with TnC guiding the separation of the TnI inhibitory domain from actin and the shift of tropomyosin that permits the creation of the actomyosin compound (Vassylyev et al., 1998; Li et al., 1999; Wang et al., 2002). The mobile domain of hcTnI comprises the C-terminal and the H4 α-helix part of the molecule (Takeda et al., 2003). It was verified that at a low level of Ca^2+^, the mobile domain of TnI cooperates with tropomyosin and the C-terminal part of actin. These relations are believed to play a vital role in the adjustment of Ca^2+^-dependent contraction and stabilization of the troponin complex on the surface of the thin filament (Galińska-Rakoczy et al., 2008; Galinska et al., 2010).

To secure the troponin complex on the actin filament, Troponin T plays a leading role in the arrangement of complex subunits and muscle contraction regulation (Tobacman, 1996; Perry, 1998). The N-terminal variable domain of the hcTnT molecule maintains central and C-terminal domains (Katrukha, 2013). Based on the latest studies on different chimeric proteins and deletion mutants of TnT, the N-terminal part of the protein manipulates the conformation of the complex and the interaction of the troponin complex with actin and tropomyosin (Wang and Jin, 1998; Chandra et al., 1999; Biesiadecki et al., 2007) and imitates the Ca^2+^ sensibility of the muscle (Sumandea et al., 2009; Gollapudi et al., 2012; Mamidi et al., 2013) and the development of maximum force of contraction (Gollapudi et al., 2013; Mamidi et al., 2013). Interacting with tropomyosin and probably actin, this C-terminal part of the molecule secures the troponin complex in the non-active state owing to the deletion of the last 14 amino acid residues hcTnT caused in the improvement of actin-activated ATPase activity *in vitro* (Franklin et al., 2012).

Troponin C consists of a short N-terminal domain formed by the first α-helix (N-α-helix) and four Ca2+ binding EF-hands that combined pairwise into the N-terminal C-terminal globular domains (Katrukha, 2013). Each EF-hand consists of two α-helices (α helices A-H) located in the Ca2+ binding loop (Takeda et al., 2003).

While the extracted troponin molecule is a moral issue for delegated structural analysis, *in vivo* troponin is a fully incorporated content of the thin filament along with tropomyosin and actin. Troponin’s collaborations with actin and tropomyosin are the basis of Ca2+-dependent regulation of the thin filament (Gordon et al., 1997), and all the mensuration of troponin regulatory function involves the whole thin filament interacting with myosin. Two essential mechanisms in the regulation by troponin components are the inhibition of the contractile interaction of myosin–actin–tropomyosin by the dissuasive activity of Troponin I and the release of the inhibition by Troponin I through the binding of Ca2+ to Troponin C. Troponin T functionally acts as an integrating component necessary for the Ca2+-regulatory action of troponin (Ohtsuki et al., 1986)

The mechanism by which troponin switches the thin filament activity in response to Ca2+ has been established for some time. At low cytosolic Ca2+ levels, the formation of the actomyosin complex is sterically inhibited by Troponin I (TnI), through its C terminus binding to actin and locking tropomyosin in a blocking position such that strong binding of actin to myosin is not allowed. Because of enhancement of the Ca2+ level, a single Ca2+ ion binds to the regulatory N-terminal cTnC site II. It initiates an intramolecular conformational shift, revealing a hydrophobic patch of NcTnC and exposing it for interaction with the cTnI switch peptide, which is also linked with a joint motion of the N-terminal TnC domain relative to the IT domain. The binding of the TnI switch peptide to the hydrophobic patch drags the C terminus of TnI afar from actin. This action permits a cooperative shift of the tropomyosin molecule across the actin surface, encountering all the myosin-binding sites on actin and thus permitting cross-bridge cycling (Marston and Zamora, 2020b). The various kinds of cTn studies are shown in Table 2.

**TABLE 2:** Several studies for Cardiac troponins.

Type of study	Number of patients	Finding	References
Retrospective	187	Elevated TnT levels in 52 patients	Guo et al. (2020)
Case Report	1	Raised serum creatinine and Troponin I level	Alhogbani (2016)
Case Report	1	Enhanced serum creatinine and Troponin T level	Hu et al. (2021)
		Troponin T was more than 10,000 ng/L. Creatine kinase isoenzyme CK-MB 112.9 ng/L	
Case Report	1	Troponin I level was 1.26 ng/ml (<0.3 ng/ml) and NT-proBNP was 1,929 pg/ml (<125 pg/ml)	Kim et al. (2020)
Retrospective	25	Elevated CRP, cTnI, D-dimer, LDH, and lactate levels	Kermali et al. (2020)
Retrospective	14,855	cTn-negative = 13,828 (N), cTn-positive = 1027 (N)	Tanboğa et al. (2021)
Retrospective	49	12% Elevated TnT levels	Zhu et al. (2020)
Retrospective	101	Almost half of whom had aN hs-TnT value fivefold more than the normal upper limit	Wei et al. (2020)
Retrospective	466	High cTnI level *N* = 168 (36.05%)	Ali et al. (2021)
Prospective	207	Elevated TnT levels, was significantly correlated with native T1	Puntmann et al. (2020)
Case Series	187	Elevated TnT levels, patients with high TnT levels had more severe respiratory dysfunction	Shi et al. (2020)
Case Report	1	Elevated levels of markers of myocyte necrosis (Troponin T level)	Inciardi et al. (2020)

Based on the latest international guidelines, classical methods are clinically dependent on cardiac troponin testing for identification and diagnosis of myocardial damage (MD) and risk stratification. However, cardiac troponin overexpressed in patients with positive risk factors for MD, and characterized by high-sensitivity immunoassays cTns (hs-cTnI and T) as gold standard laboratory techniques in adults and pediatric age (Clerico et al., 2021a). Additionally, hs-cTn approaches are capable of monitoring myocardial renewal and remodeling mechanisms, and can be used to quickly identify individuals who are mostly at risk of developing symptomatic heart failure, perhaps leading to earlier diagnosis and a better prognosis (Clerico et al., 2021b).

## 5 Diagnostic Value of Troponin in COVID-19

Cardiac injury is frequently encountered in patients with COVID-19 and is associated with increased risk of death (Wibowo et al., 2021). Cardiac complications include the development of incident heart failure, acute coronary syndrome (ACS), and arrhythmia, all of which are associated with elevation in cTn (Park et al., 2017). Elevated troponin may signify myocardial damage and is predictive of mortality. However, the prognostic performance of troponin and whether its value is affected by various comorbidities that may be present in patients with COVID-19 are not known (Wibowo et al., 2021). In addition to causing pneumonia (the main complication of the infection), SARS-CoV-2 may induce a direct damage to the heart: on the one hand, causing a myocardial infection (myocarditis), with significant impairment of cardiac contractility; on the other hand, it might affect the pericardium (pericarditis) with the formation of an effusion that may also impair cardiac function (Chen et al., 2020). In case of viremic phase, the mechanism by which the virus might attack heart cells could be related to the elective affinity between the viral spike proteins of SARS-CoV and type 2 angiotensin-converting enzyme receptor (ACE-2), which is well represented on myocardial cells. Another hypothesis is that the virus may migrate from the lung with infected macrophages to the myocardium (Piccioni et al., 2020). ACE-2 is also present on the endothelial cells of the vessels, so theoretically, acute vasculitis (inflammation of the vessels) of the intra-myocardial vessels could also occur, which would end up causing ischemic damage (Hanff et al., 2020) .

Myocardial damage could also be caused by severe general inflammation. This leads to the release of abundant quantities of inflammatory substances (cytokine storm), with a toxic effect on the heart muscle, thus compromising its function (Tveito, 2020). It is also possible that, in some cases, the adrenergic hyperactivation following respiratory distress and, possibly, the psychic stress related to the condition cause a ventricular dysfunction typical of the Tako-Tsubo syndrome, or an acute myocarditis that presents itself as a Tako-Tsubo syndrome (Sala et al., 2020).

Finally, it has been shown that cardiac function can be seriously compromised due to the severe infectious state in patients with known heart failure, myocardiopathy, or serious valvular diseases (Dong et al., 2020). In all these manifestations of heart damage, the evaluation of cardiac biomarkers such as troponin is essential, above all to highlight an early diagnosis of cardiac involvement, to guide a possible prognosis, and to present a helpful follow-up (Piccioni et al., 2020). As mentioned above, troponin increase correlates with the severity of infection (Metkus et al., 2017).

A meta-analysis carried out calculating the standardized mean difference (SMD) and 95% confidence interval (95% CI) of cTnI or cTnT values in COVID-19 patients with or without severe disease has shown that cTnI concentration is only marginally increased in all patients with SARS-CoV-2 infection, whereby values exceeding the 99th percentile in the upper reference limit (URL) can only be observed in 8%–12% of positive cases (Lippi and Plebani, 2020c).

In an observational cohort study of patients with COVID-19, all of the following biomarkers were measured: Troponin I, B-type natriuretic peptide, C-reactive protein, ferritin, and d-dimer. Among the tested biomarkers, Troponin I ≥0.34 ng/ml was the only independent predictor of 30-day mortality. Use of a simple risk score, which incorporates troponin levels, age, and presence of hypoxia on presentation, can help stratify patients at risk for in-hospital mortality associated with COVID-19 (Manocha et al., 2021).

A retrospective analysis was carried out on 54 subjects admitted to Tongji hospital in February 2020. Patients with or without myocardial damage, defined with three times higher serum cardiac troponin value, were analyzed and compared. During hospitalization, 44% of cases (*n* = 24) were complicated by myocardial damage and 48% (*n* = 26) died in the hospital. Mortality was significantly higher in patients with myocardial damage than in patients without myocardial damage, and this correlated with the values of troponin, C-reactive protein, and pro-BNP. This study also confirms that the involvement of myocardial tissue in COVID-19 disease correlates with the severity of the clinical picture. COVID-19 patients with severe respiratory failure and myocardial damage have a significantly higher risk of in-hospital mortality. In addition, the study suggests that it is important to monitor patients with high troponin values at the first check with the serial dosages of this biomarker to understand the evolution of the myocardial injury during hospitalization for COVID-19 patients (Piccioni et al., 2020) .

Evidence of COVID-19-associated increases in circulating cardiac Troponin T (cTnT) and cardiac Troponin I (cTnI) above the 99th percentile reference limit is emerging in the literature (Wang et al., 2020a; Liu et al., 2020d; Ruan et al., 2020).

Elevated serum troponin levels on admission statistically correlated with mortality in COVID-19 patients (Al-Zakhari et al., 2021). In a retrospective cohort analysis, cTnI was significantly elevated in 54 subjects who died compared with 137 survivors (median [IQR] cTnI 22 [5.6–83.1] ng/L vs. 3 [1.1–5.5] ng/L, *p* ≤ 0.0001) (Zhou et al., 2020).

The principal pathophysiology indicates a cardio-inflammatory response, as many significantly ill COVID-19 patients determine concomitant elevations in acute phase reactants such as CRP and the natriuretic peptides. This action may present clinically as fulminant myocarditis (Gaze, 2020).

In one case report study, a 37-year-old man presented with a 3-day history of chest pain and dyspnea. Electrocardiographic data implied an ST-segment elevation acute MI, and cTnT was substantially elevated at >10,000 ng/L (99th percentile reference limit <14 ng/L), with simultaneous elevations in CK and B-type natriuretic peptide. The appropriate working diagnosis was ACS. Subsequent CT coronary angiography discovered no verification of coronary stenosis. A sputum sample was assessed for 13 viral nucleic acids, of which only coronavirus was positive. The diagnosis changed to coronavirus fulminant myocarditis with cardiogenic shock and pulmonary infection. The patient was successfully handled with glucocorticoid and human Ig; cTnT diminished to 220 ng/L from 1 to 3 weeks (Hu et al., 2021).

## 6 Development of Biosensors for Cardiac Troponin in COVID-19

Biosensors are devices designed to detect and quantify target biomarkers in certain diseases and thus aid in the early prediction of these complicated disorders (Nakamura and Karube, 2003; Wang et al., 2005). Their prime principle is similar to antigen–antibody interactions, whereby agents of interest like an enzyme, antibody, or DNA/RNA molecules are coated over the transducer surface, and upon interaction with the target substance or biological elements of interest, they generate quantifiable signals (Prieto-Simón et al., 2014; E Wang et al., 2011; Pei et al., 2013). However, both antibody and antigen provided high specificity, sensitivity, and applicability in humans (Sivasubramanian et al., 2009). The tendency for fabrication of biosensors is a vital needed process to resolve limitations encounter classical bioassays, critical care overload with a wide limit of detection (LOD) range, rapid turnaround time, high stability, reliable detectability for undiagnosed or asymptomatic cases, low costs and compatible with sustained lab quality schemes.

Quantification of cardiac troponins is among the most vital biomarkers of cardiovascular disorders especially MI. Due to their high specificity and sensitivity, cardiac troponins are considered vital markers of coronary events (Spyracopoulos et al., 1997; Takeda et al., 2003; Babuin and Jaffe, 2005). Usually, the circulating troponin level is very low in normal patients and is not usually detectable; however, in acute MI, its level increases within 24 h of myocardial events (Antman et al., 2000). Biosensor technologies are capable of early detection of troponin in human biological fluids and thus are very important tools in survival against cardiovascular fatalities (Abdolrahim et al., 2015).

### 6.1 Types of Cardiac Troponin Biosensors

**FIGURE 1:**
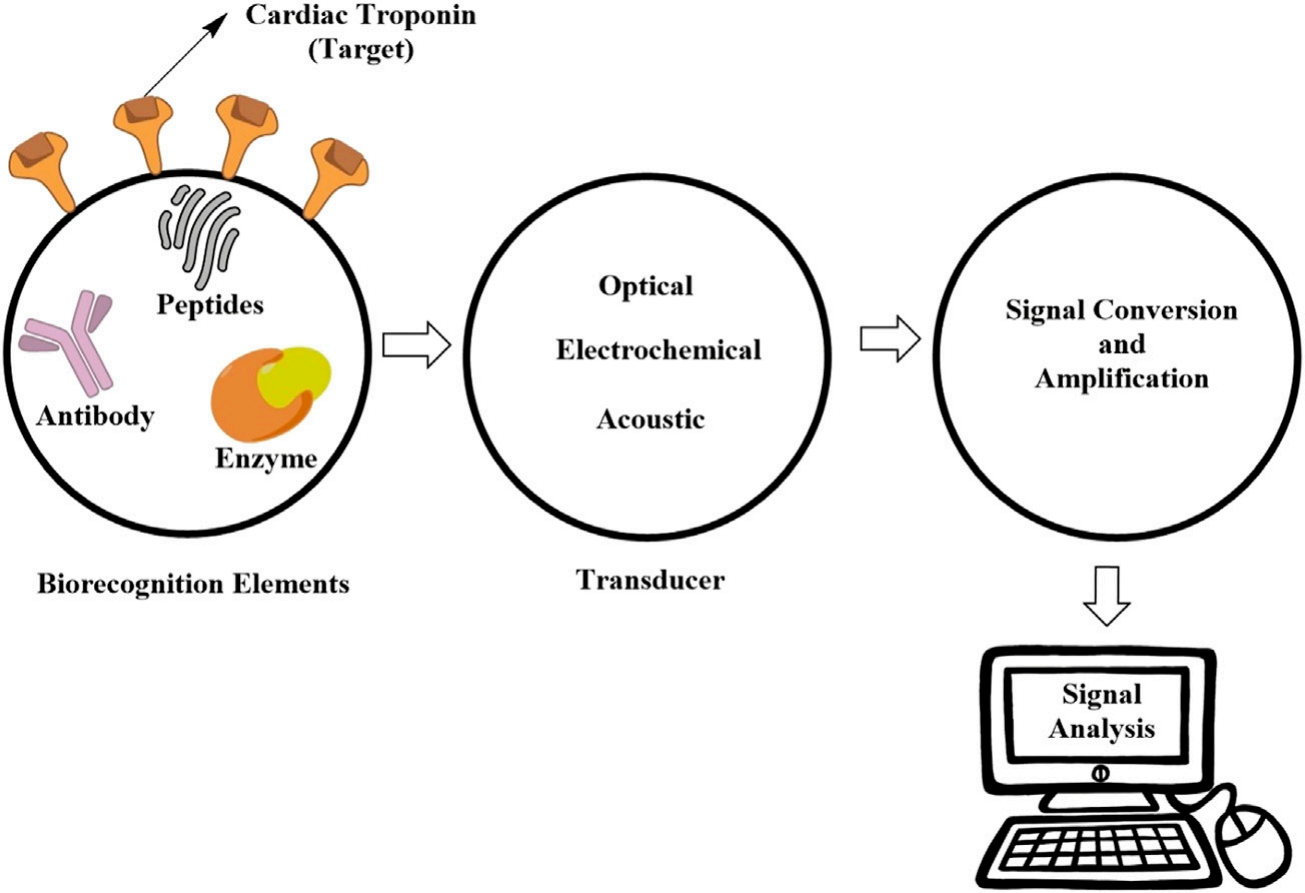
Mechanisms underlying the primary function of biosensors.

Many developed troponin biosensors are different from each other according to transduction mechanisms and broadly grouped into electrochemical, optical, and acoustic detectors. Electrochemical detectors include amperometric, impedimetric, potentiometric, as well as conductance-dependent detectors as in Figure 1. Likewise, optical detectors are exemplified by fluorescence- and chemiluminescence-dependent detectors, surface plasmon resonance (SPR)-based detectors, and surface-enhanced Raman spectroscopy (SERS) detectors. Yet, another group of acoustic detectors includes surface acoustic wave (SAW)-dependent detectors and quartz crystal microbalance (QCM)-dependent sensors, as summarized in Table 3.

**TABLE 3:** A detailed comparison between cardiac tropobiosensors used in lab diagnosis.

Biosensor name	Used technique	Detection limit	Pros	Cons
ZnO or porous reduced-graphene oxide (rGO) nano-sensor utilizes R-S-H linker and Troponin antibodies *via* EIS. Liu et al. (2020d), Manocha et al. (2021)	Electro-impedimetric sensors (EIS)	0.07 ng/ml	• Detection of both Troponin I and Troponin T in pico-amounts	• The biocompatibility and non-toxicity of graphene nanomaterials are still not confirmed
			• Highly sensitive, cost-effective, and selective	• Impedimetric sensors suffer from hesitations in resistivity
• *Graphene quantum dots (GQDs) and polyamidoamine (PAMAM)-modified gold electrodes.* Ruan et al. (2020)	Electro-amperometric sensors (EAS) based on voltammetry	20 fg/ml	• Highly stable and sensitive electrodes	Testing errors in the preclinical stages
			• Assimilation of targeted immunoreactions without interferences	
		50 ng/ml	• Uses tiny amounts of patient serum sample and gives results in a few minutes	
• *Abbott- immuno i-STAT handheld.* Al-Zakhari et al. (2021)			• Widely used and considered as one of the successful POC tool for detection of cTns	
Polypyrrole-coated ISFET sensor. Gaze (2020)	Electro-potentiometric sensors (EPS)	0.01 ng/ml	Robust and ultrasensitive with wide detective range	Delayed results up to 20 min
Silicon nanowire-based sensor. Nakamura and Karube (2003)	Electro-conductometric sensors (ECS)	1 ng/ml	• Detect serum cTns up to 1 fg/ml	Less stable due to the effect of salt concentration in the buffer and the length of the wire used
TiO_2_ nanotube array (TNTA). Wang et al. (2005)	Fluorescence-based sensors (FBS)	0.1 pg/ml	• Cheaper, sensitive, increased surface area with high compatibility and applicability	• Needs high temperature
			• Detect serum cTns up to 100 pg/ml	• TNTA affected by presence of impurities and changes in pH
				• The power of detection is a function on nanotube length and thickness
				• Applied only on 10% diluted serum
Gold nanoparticles (AuNPs)-modified TiO_2_ nanotube array (TNTA). Prieto-Simón et al. (2014)	2.2 pg/ml	Swift detection with accuracy	Low detection limit than ELISA
Classical sandwich ELISA. E Wang et al. (2011)	Chemiluminometric immunosensors (ELISA)	0.02 ng/ml	Reliable assays	• Slow turnaround time about 20 min
				• Not cheap
ELISA-on-chip biosensor based on cross-flow chromatography for detection of antibodies. Pei et al. (2013)		0.01 ng/ml	• Swift detection up to 30 s	• Not commonly used
			• Suitable for POC	• The cost per test is still not cheap enough
			• Cheaper and more sensitive than conventional colorimetric assays	
Poly(dimethylsiloxane) (PDMS)–AuNPs composite-based biosensor. Sivasubramanian et al. (2009)		0.01 ng/ml	Precise, easy fabrication, and high stability	• Difficulty in labeling, expensive, and bulky
				• Detection time not less than 20 min
				• Still under experiment
Ru-PAMAM/AuNPs-based electrochemiluminescence (ECL). Spyracopoulos et al. (1997)	Electrochemiluminescence (ECL)	12 fg/ml	• Better sensitivity, specificity, stability, and reproducibility	Still being tested
			• Label-free method	
Surface enhanced Raman spectroscopy (SERS)-based competitive immunoassay. Babuin and Jaffe (2005)		33.7 pg/ml	• Total detection time is 7 min	• Metallic coated nanoparticles may be toxic *in vivo* overtime
			• High specificity and stability with sharp bands	• Imaging problems due to insufficient light wavelength used to penetrate body tissues
				• Low precision due to scattering possibilities
Localized surface plasmon resonance (LSPR)-based nanosensor. Antman et al. (2000)		250 × 10^–^ 6 ng/L	• High sensitivity, applicability, and reproducibility	• Presence of any other analytes in the solution may lead to overlapped peaks in the infrared region
			• Easily detect cTnT in asymptomatic cases	• Limited penetration to 100 nm makes it a bad choice for large molecules
			• Low cost	• Unable to detect cTnT from cell extracts
			• Label-free method	
Quartz crystal microbalance (QCM) -based sensor. Abdolrahim et al. (2015)	Acoustic sensors	5 ng/ml	Sensitive compared to EIS for detection of cTnI	Needs delicate control in pressure and temperature
Surface acoustic wave (SAW)-based sensors. Lomant and Fairbanks (1976)				Still under evaluation

### 6.2 Electrochemical Sensors

The basic principle of these detectors is based on a change in the potential difference or current/impedance after immunological reactions taking place at the electrode surface. So, these types of biosensors have numerous biological molecules like various metabolites, enzymes, nucleic acid, and DNA acting as detection probes. These sensors are highly sensitive, cost-effective, selective, as well as reliable regarding their results. They are further grouped into the following:

(1) Impedimetric Sensors

These sensors quantify the change in resistivity value after potential is applied across the electrodes. In the presence of some electrolytes at the surface of the specialized impedimetric sensor, upon application of potential to the electrode, polarization is observed. Subsequently, charges move to the electrode surface, thus forming an electric double layer. This double layer is essential since it is a pocket of charges and substantially changes upon antigen–antibody interaction at the electrode surface. Various cross-linking polymers are used to adequately immobilize the antibodies at the electrode surface (Lomant and Fairbanks, 1976). These polymers contain the R-S-H group upon which they bind with the gold electrode whereas another CO-NHS group is available for making bonds at the other end. When interacting with an antibody, the bond breaks and an amine link is formed with the antibody. Various types of linkers are used based on the type of electrode and type of substrate (Choi et al., 2000; Swaim et al., 2004). For instance, ZnO nano-sensor utilizes an R-S-H linker and α-cTnT antibodies *via* EIS to detect cardiac troponins. This detector can measure both the Troponin I and Troponin T in picograms inside the human blood (Shanmugam et al., 2017). A similar detector using porous graphene oxide substrate was developed by Kazemi et al. (2016).

(2) Amperometric Sensors

The device works on the same basic principle as the previous one, whereby specific antibodies are immobilized at the surface of electrodes, and upon reaction with antigen, changes in the current are measured (Stetter and Li, 2008). These sensors are different from the previous one because impedimetric sensors record resistivity changes. In amperometric sensors, change in current is measured after antibody reaction with a specific antigen or biomolecule. The electrodes also contain redox probe or chemical mediator-labeled antibodies, which assist in quantifying changes in current at working electrodes and thus provide beneficial information about the antigen–antibody binding mode after applying a specific potential bias to the electrode. Subsequently, alteration in the current relative to the reference electrode is calculated. In these sensors, signal detection is usually done through cyclic voltammetry. Most commonly, sensing electrodes are made of carbon nanotubes owing to their flexible electrochemical properties including better electrical conductance, coherence with other materials, and mechanical strength (Gomes-Filho et al., 2013). Having a high electron transport rate, the reaction rate for the majority of antigen–antibody reactions is faster with higher sensitivity and better performance. Typically, i-STAT handheld is an amperometric sensor developed by Abbot United States and worked on the same immunoassay-dependent approach. This sensor can detect cTnI from blood samples (17 µl) in concentrations as low as 0–50 ng ml^−1^ in a few minutes *via* a disposable cartridge. This biosensor is very useful clinically for detecting MI and the prognosis of the disease (Steinfelder-Visscher et al., 2008).

(3) Potentiometric Sensors

These sensors assess any potential difference between the set of electrodes separated from each other *via* a semi-permeable membrane. Changes in potential difference escalate due to either pH changes or oxidation–reduction reactions pertinent to the electrode surface (Bakker and Pretsch, 2005). When interactions happen between antigen adsorbed at the electrode–electrolyte interface and immobilized captured probe, relevant changes in the potential difference arise (Novell et al., 2012). The change in potential is proportional to the extent of antigen binding with capture probe. Light addressable potentiometric sensors (LAPSs), pH electrode-reliant glass ion-selective electrodes (ISEs), and solid-state ion-selective field-effect transistors (ISFETs) are examples of this type of sensor. The modified versions of ISE sensors are field-effect transistor sensors and are mostly preferred owing to their better immuno-sensing capabilities.

Further, Cambridge researchers developed a new polypyrrole-coated sensor as an example of ISFET capable of detecting cardiac troponins (Purvis et al., 2003). This sensor is superior for having the ability to immuno-separate during analysis; the pyrrole layer at the electrode surface is polymerized *via* potentiodynamic electropolymerization cascade, making the sensor more robust and highly sensitive. Potential difference develops as a result of receptor–target interactions, and a complex is formed with the polypyrrole layer found on the electrode surface and is proportional to the amount of cardiac Troponin I molecules binding to its monoclonal antibodies (Palan et al., 1999).

(4) Conductance-Dependent Sensors

These types of sensors measure biological molecules on the basis of changes in the conductance values of solutions. Firstly, specific voltage is applied to a sensor that acts as a microfluidic channel coated with biomolecules like antibodies, thus generating a baseline. This baseline provides initial measurement values for later comparison when the conductance changes after reaction with an antigen-containing solution (Lee et al., 2009). Antigen–antibody interactions considerably change the quantity of current required for detection, which is subsequently construed to measure the solution conductance. Researchers developed a silicon nano-wire-dependent sensor working on the same principle and capable of detecting cardiac Troponin T in blood as low as 1 ng ml^−1^ (Chua et al., 2009).

### 6.3 Optical Sensors

The basic principle of these biosensors is to detect any changes in the input light frequency or changes in its polarization phase after immunological reaction. Hence, they are also named as fluorescence, luminescence, surface plasmon resonance (SPR), and colorimetric sensors (Fan et al., 2008).

(1) Fluorescence-Reliant Sensors

The first-ever fluorescence biosensor was reported in 1941 (Coons et al., 1941), where the target analyte labeled with fluorescent dye or probe is allowed to react with a biomolecule leading to a change in the fluorescence intensity. Later on, a more efficient and modified “point-of-care” biosensor using TiO_2_ nanotube arrays was developed by Kar et al. (2012). Though having high sensitivity, these sensors are associated with a tedious labeling process and difficult quantitative analysis due to signals emitted from fluorophores on molecules (Cox and Singer, 2004). Because of this, ellipsometry-based biosensors are usually preferred. A silicon optical sensor with a dielectric spin applied over the top of the silicon substrate and subsequently functionalized for cardiac Troponin I detection is also developed. However, the LOD of these sensors is relatively low compared to electrochemical detectors and cannot quantify cardiac Troponin I in pure serum (Diware et al., 2017a). Further, photo-electrochemical sensors also have the same basic working principle, but they are coupled with a photo-responsive device in addition to the normal immuno-sensing components. The target antibody is usually immobilized at the electrode surface and response is observed *via* laser light. Photo-current is usually detected proportionally to antigen (troponin) concentration. When an immunological reaction between antigen and antibody occurs, this leads to decline in photo-current. The LOD of these sensors is quite high and comparable with electrochemical sensors (Tan et al., 2017).

(2) Chemiluminometric Sensors

In these calorimetric sensors, the quantity of target biomolecules is assessed from the quantity of light absorbed by the chromogenic agent at a specific wavelength. ELISA is a typical example of these calorimetric sensors that again work on immunological reactions’ principles (Cho et al., 2009). Initially, a biomolecule acting as antigen is complexed with a primary antibody that is subsequently linked to an enzyme-linked secondary antibody. The biomolecule of interest is quantified from the activity of the conjugated enzyme generated by the product. A modified version of this concept is “ELISA on chip” for the quantification of cardiac Troponin I (Cho et al., 2009). Among the novel strategies is the use of nano-materials whereby nano-carriers are loaded with an extra quantity of enzymes, which enormously increase the quantity of signal-emitting molecules and thus improve signal amplification (Li et al., 2013). For instance, a PDMS–gold composite biosensor was reported by Wu et al. in 2010 for the detection of cardiac Troponin I (Wu et al., 2010). Additionally, these nano-particles (i.e., AuNPs) are well-functionalized substrates for biological target molecules including antibodies, antigens, and enzymes. So, the combination of polydimethylsiloxane (PDMS) with AuNP_S_ is ideal for developing calorimetric sensors.

(3) Surface-Enhanced Raman Spectroscopy-Dependent Sensors

The multiplex and highly sensitive SERS biosensors have significantly improved the issues associated with the primitive Raman spectroscopy method like low accuracy due to less scattering efficiency (Campion and Kambhampati, 1998). The basic principle of SERS is excitation of electrons from a roughened metallic surface *via* laser or other electromagnetic radiation, which subsequently starts oscillation, having the same wavelength of the incident radiation. Thus, enrichment in electric field occurs due to addition of a secondary electric field to the already present electric field. When the electrons’ movement is restricted at a particular oscillation frequency, resonance is experienced in the incident field. The resulting resonance is vital in the detection of biological molecules of interest (Vo-Dinh, 2008). Signal detection for biological targets can be further improved *via* addition of nano-scale roughness and specialized coating of the metallic surface. One SERS biosensing technique for cardiac Troponin I utilizes a magnetic bead surface coated with immobilized antibodies having nano-tags for binding with target molecules. This SERS device has comparatively high sensitivity (LOD 33.7 pg ml^−1^) than other techniques (Zhang et al., 2011).

(4) Surface Plasmon Resonance-Dependent Sensors

Since its inception 1983 by Liedberg, SPR-based sensors have been widely used to quantify biological molecules including detection of cardiac troponins (Liedberg et al., 1983). These sensors are capable of quantifying equilibrium constants for reactions like protein–protein interactions, protein interactions with DNA molecules, and other ligands. The sensor consists of a glass prism coated with immobilized biological molecules like antibodies and the molecules to be detected allowed to flow through it. Subsequent to immunological reaction (i.e., antigen–antibody reaction), the light intensity changes proportional to the amount of target molecules (Englebienne et al., 2003). Researchers in United States developed an extremely sensitive SPR-type sensor using LSPR (localized surface plasmon resonance) capable of detecting very minute quantities (10^−18^ M) of cardiac troponins in biological fluids like blood, urine, and serum (Liyanage et al., 2017). A modified LSPR using human Troponin I binding peptide, applied on gold nano-rods was also developed (Tadepalli et al., 2015).

### 6.4 Acoustic-Dependent Sensors

These sensors are constructed on the basic theory of mass evaluation *via* piezoelectric crystals. For instance, when immunological reactions occur causing the formation of complex at the electrode surface, the mass changes cause a shift in the crystal frequency, thus generating an electrical signal.

(1) Quartz Crystal Microbalance

The QCM contains quartz crystal and the device utilizes the piezoelectric properties of the same crystal and thus measure variation in mass as a result of changes in resonant frequency as explained by the Sauerbrey formula; ∆f = −2f^2^∆m/Aρυ.

QCM sensors are in clinical use to quantify cardiac troponins. The sample is applied at the sensor and the surface of electrodes pre-loaded with the target immobilized antibodies causing mass loading of the sensor after the reaction. Researchers have developed a cost-effective QCM sensor for the analysis of cardiac Troponin T. When a shift occurs in the frequency, it generates electric signals corresponding to the mass accumulating at the electrode surface (Wong-ek et al., 2010).

(2) Surface Acoustic Wave-dependent Sensors

These sensors also operate based on the same principle of QCM, where interdigitated transducer electrodes are coupled with piezoelectric crystal surface. The electrical signals are converted by transducers to polarized transversal acoustic waves that move across the piezoelectric crystal. Accumulation of mass over the piezoelectric crystal causes a shift in frequency of the acoustic waves generated by subsequent biosensing (Pohl, 2000). Examples of these sensors are Rayleigh-SAW, Love-wave, and Lamb-wave sensors, which are commonly used to assess cardiac Troponin I in biological samples (Priya et al., 2015). A Love-wave-type SAW sensor was reported previously to detect cardiac Troponin I, whereby immobilized antibodies on AuNPs react with cardiac Troponin I antigens and thus a response is sent to the sensor (Lee et al., 2011).

### 6.5 Tailoring Cardiac Troponin Detectors for COVID-19 Patients

Baker et al. developed the LOCKR system to produce a biosensor that utilizes light to indicate the presence of the target molecule. Initially, LOCKR proteins are present in a “closed” state and unable to emit light. When, the target molecule is present, it will bind with a specific region of the sensor, switching the LOCKR to the “open” state and emit light, which is easily recorded. The key feature of the LOCKR system is that it can easily be adapted to sense a range of targets, because the target binding region can be swapped without affecting the rest of the system. The biosensor was reliable in detection of SARS-CoV-2 virus in tiny amounts as 15 p.m. in 2 min and may able to detect a range of targets dependent on the benefit of the LOCKR system (Quijano-Rubio et al., 2021).

Surprisingly, a newly sensitive microfluidic biosensor with mesoporous nickel vanadate hollow-nanosphere modified chitosan (Ch-Ni_3_V_2_O_8_) template was designed for detection of cTnI levels in patient serum samples depending on the high redox activity and biocompatibility of the Vanadium. However, this matrix worked against cTnI antibodies with a detection limit to 5 pg/ml with high sensitivity, selectivity, and reproducibility (Singh et al., 2019).

Xian et al. built a hand-made modularized Si MOSFET-based biosensor able to detect SARS-CoV-2 spike proteins in saliva to 100 Fg/ml and cTnI levels down to 100 pg/ml. This platform is recommendable for POC at emergent events and pandemic crises due to its cheaper cost and disposable sensor units (Xian et al., 2020). Recently, a smartphone app joined with an autonomous capillary microfluidic chip (ACMC) and self-aligned on-chip focusing (SOF) lenses to quantify serum cTnI levels in 12 min with 78–94 pg/ml detection limits using 100 μl of sample, based on the sandwich immunofluorescence principle. However, this creative platform is possibly used for POCT field in resource-limited settings (Liang et al., 2019). Boonkaew et al. designed a successful model of graphene oxide-modified carbon electrode stencils printed on an ePAD for detecting CRP, cTnI, and procalcitonin (PCT) using the square wave voltammetry (SWV) principle. Their results showed significant LOD values for cTnI about 0.16 ng/ml with *R* ^2^ > 0.99 and RSD < 5% (Boonkaew et al., 2021). Synergistically, a ZnO-NPs-based FET biosensor showed a diagnostic efficiency with LOD of 3.24 pg/ml in the detection of serum cTnI (Boonkaew et al., 2021).

Another fascinating model that consists of a Tetrahedral DNA (TDs) aptamer built in HCR and Au/Ti3C2-MXene amplified units on EC/ECL biosensors detected cTnI levels in blood samples of COVID-19 critical cases with LODs of 0.04 or 0.1 fM (Sandoval et al., 2020). Indeed, reaching out to models of biosensors of sufficient sensitivity and reliability will contribute to predict CVD-associated COVID-19 complications at a time early enough for clinical interventions.

## 7 Challenges and Perspective

As new cases are being recognized during the COVID-19 pandemic, identification of clinical and diagnostic presentations is being refined. Cardiac biomarkers, specifically natriuretic peptides and cTn, are usually promoted in COVID-19 patients. In pathologies of majority of diseases, the elevation of cTn is related to disease severity and poor prognosis (Fang et al., 2020). The method of using sequential cardiac troponin can make risk classification easy, helps make decisions about how and when to use preclinical manifestations, and informs stage grouping and disease phenotyping along with hospitalized COVID-19 patients (Sandoval et al., 2020).

The principally significant roles of the cardiac troponins, cTnI and cTnT, are as diagnostic gold standard and the most specific and sensitive biomarkers for cardiac injury and acute MI and in categories threats in acute coronary syndrome and myocardial necrosis (Apple et al., 2012). Recent studies have revealed that sequential analysis of cT in patients with chronic heart failure is able to give more sufficient prognostic data. Patients with a high level of troponin concentrations have a worse prognostic fate than others (Masson et al., 2012), for instance, higher decreasing ejection fraction (cardiac output), lower systolic blood pressure, higher rate of in-hospital mortality and acute decompensated heart failure, and higher re-hospitalization rate in both ischemic and non-ischemic heart failure. Regardless of the cause of heart failure, increased cTn is an autonomous predictor of poor consequences in individuals with heart failure outside of the setting of acute MI. In clinical studies, the influence on therapy and follow-up has yet to be determined (Peacock et al., 2008; Catarino et al., 2021).

The release of cTn in the peripheral blood starts in 3–24 h, and maximum rates of up to 10–20 h for cTnI and more than 15 to 120 h for cTnT return to original levels after 10 days (cTnI) and 14 days (cTnT) (Danese and Montagnana, 2016). The European Society of Cardiology (ESC) and the American College of Cardiology (ACC) both consider troponin increases to be critical in the diagnosis of AMI. To eliminate false-positive outcomes, the ESC/ACC set a cutoff value for diagnosing acute MI as an increased value of cardiac biomarkers over the 99th percentile of the upper reference limit. This percentile can be changed depending on the patient’s age, sex, or nationality (Catarino et al., 2021). A retrospective study on diverse analytic situations showed that gender can affect the results of cTnI, which may boost the sensitivity in women and specificity in men. Finally, their data confirm that a diagnosis of MI still relies on clinical opinion and evaluating cTn levels should be seriously assessed and should consider the clinical manifestations (Eidizadeh et al., 2021). Clinicians should be able to interpret cardiac biomarkers in the same manner as they comprehend blood count reference ranges, in order to get the most out of greater sensitivity, quicker decision paths, and improved risk prediction (Alaour et al., 2018).

Notice to importance of cTn, researchers have been following the safe, noninvasive and more sensitive and specific techniques with the least cost, time consuming and commercially accessible for a minimal detection amount of troponin in the blood and recently in saliva. Nowadays, there are numerous high-sensitivity biosensors published in the literature for measuring troponins in serum or whole blood within 30 min, with LOD as low as a few pg/ml (Liu et al., 2016) or even less than 1 pg/ml (Diware et al., 2017b), and only three biosensors were effectively defined in literature for recognition and quantification of troponins in saliva. All three salivary troponin biosensors are detected as type cTI: bead-based ELISA with spectrophotometric detection at 450 nm (Park et al., 2012), electrochemical–differential pulse voltammetry (DPV) (Chekin et al., 2018), and fluorescence spectroscopy in the 220–350 nm range (Rezaei and Ranjbar, 2017).

Furthermore, the pathophysiology of cardiovascular disease (CVD) and COVID-19 in acute steps is similar and driven by several biological procedures, including oxidative stress, inflammation, interaction between hormone and nervous system activation, or myocyte injury. However, the collective use of various biomarkers may obtain complementary prognostic information and may provide a better device to reduce mortality rates of COVID-19 based on coronary artery disease (CAD) involvement. In a patient with high-troponin levels besides increased levels of CRP, adiponectin and soluble intercellular adhesion molecule (sICAM)-1 were significantly raised in both blood and saliva after acute myocardial infraction (AMI). In conclude, all of the world healthcare services is attempting to prevent and early diagnosis of CAD and AMI for well prognostic future and effective treatment specially during the COVID-19 pandemic. Therefore, the early detection of AMI has a critical and cost-effective role in patient survival. The ability to detect a TN quickly and accurately in a small volume of body fluids (serum and saliva) and at an extremely low concentration (less than 24 pg/ml in a blood sample) is required for the development of a POCT TN detection device. Various biosensors and devices have been presented so far to detect TNs. These biosensors will create new chances for rapid recognizing with low LOD and high precision, relying on similar pathophysiology of cTn in COVID-19 and CAD (Henry et al., 2020a; Lippi and Plebani, 2020b).
